# Medication Adherence, Burden and Health-Related Quality of Life in Adults with Predialysis Chronic Kidney Disease: A Prospective Cohort Study

**DOI:** 10.3390/ijerph17010371

**Published:** 2020-01-06

**Authors:** Wubshet H. Tesfaye, Charlotte McKercher, Gregory M. Peterson, Ronald L. Castelino, Matthew Jose, Syed Tabish R. Zaidi, Barbara C. Wimmer

**Affiliations:** 1 Pharmacy, School of Medicine, College of Health and Medicine, University of Tasmania, Hobart 7005, Tasmania, Australia; g.peterson@utas.edu.au (G.M.P.); barbara.wimmer@utas.edu.au (B.C.W.); 2 Menzies Institute for Medical Research, Hobart 7000, Tasmania, Australia; charlotte.mckercher@utas.edu.au; 3 Sydney Nursing School, The University of Sydney, Sydney 2006, New South Wales, Australia; ronald.castelino@sydney.edu.au (R.L.C.); Matthew.Jose@utas.edu.au (M.J.); 4 Royal Hobart Hospital, Hobart 7000, Tasmania, Australia; 5 School of Healthcare, University of Leeds, Leeds LS2 9JT, UK; s.t.r.zaidi@leeds.ac.uk

**Keywords:** chronic kidney disease, medication adherence, health-related quality of life, medication regimen complexity index, medication burden

## Abstract

This study examines the associations between medication adherence and burden, and health-related quality of life (HRQOL) in predialysis chronic kidney disease (CKD). A prospective study targeting adults with advanced CKD (estimated glomerular filtration rate (eGFR) < 30 mL/min/1.73 m^2^) and not receiving renal replacement therapy was conducted in Tasmania, Australia. The actual medication burden was assessed using the 65-item Medication Regimen Complexity Index, whereas perceived burden was self-reported using a brief validated questionnaire. Medication adherence was assessed using a four-item Morisky-Green-Levine Scale (MGLS) and the Tool for Adherence Behaviour Screening (TABS). The Kidney Disease and Quality of Life Short-Form was used to assess HRQOL. Of 464 eligible adults, 101 participated in the baseline interview and 63 completed a follow-up interview at around 14 months. Participants were predominantly men (67%), with a mean age of 72 (SD 11) years and eGFR of 21 (SD 6) mL/min/1.73 m^2^. Overall, 43% and 60% of participants reported medication nonadherence based on MGLS and TABS, respectively. Higher perceived medication burden and desire for decision-making were associated with nonadherent behaviour. Poorer HRQOL was associated with higher regimen complexity, whereas nonadherence was associated with a decline in physical HRQOL over time. Medication nonadherence, driven by perceived medication burden, was prevalent in this cohort, and was associated with a decline in physical HRQOL over time.

## 1. Introduction

Medication adherence is the primary determinant of treatment success, yet 43% to 78% of people receiving medications for chronic diseases are nonadherent to medical treatment. [1] The reported prevalence of medication nonadherence in chronic kidney disease (CKD) also varies considerably; 12%–53% in stage 3 to 4 CKD and 21%–74% in end-stage kidney disease (ESKD) [2,3,4]. Medication adherence is particularly relevant in people with CKD, given its potential importance in slowing disease progression and, therefore, improving health outcomes. Poor adherence to antihypertensive medications in CKD, reported in nearly one-third of patients, is associated with uncontrolled hypertension [5,6]. Research also indicates that nonadherence to cardiovascular medications at the predialysis stage is an independent predictor of post-dialysis mortality in people with advanced CKD [7].

Patient-centred outcomes, such as health-related quality of life (HRQOL), are important measures that capture patients’ perspectives and experiences about their functionality and wellbeing [8]. These outcome measures are particularly relevant in patients with advanced CKD, as they inform treatment goals and modalities [9]. Nevertheless, there is limited data on patient-centred outcomes in people with advanced CKD [9,10,11], particularly in those not receiving renal replacement therapy [12]. More importantly, the relationship between HRQOL and medication-related factors, such as medication burden and adherence, is relatively under-examined in this patient group [13].

The actual and perceived medication burden can be assessed in different ways, including the complexity of medication regimens and the number of medications used. Medication regimen complexity and the number of medications would be expected to influence adherence, although the findings on this subject are not consistent [14]. In patients with CKD, the association between medication regimen complexity and adherence is inconclusive [10,11]. Moreover, despite the high medication burden in patients with advanced CKD, evidence is lacking on medication-related factors and patient-centred outcomes in predialysis CKD.

This study aimed to (i) identify factors associated with medication burden (perceived and actual), (ii) examine the association between medication burden (actual and perceived) and adherence in adults with predialysis CKD, (iii) examine the association between HRQOL and actual medication burden, and (iv) evaluate the relationship between medication adherence and change in HRQOL over time.

## 2. Materials and Methods

### 2.1. Study Design and Population

This analysis utilised data from the Tasmanian CKD study, a prospective cohort of Tasmanian adults aged ≥18 years with advanced CKD (based on a single estimated glomerular filtration rate (eGFR) reading of <30 mL/min/1.73 m^2^ in the 3 months prior to recruitment) and not receiving renal replacement therapy. A detailed description of the rationale, design and preliminary results has been published previously [15]. Participants were recruited through their treating physicians and attended a baseline clinic between February 2016 and September 2018. Individuals with at least one medication and who agreed to participate in an additional medication interview were included in the current analysis.

At baseline, participants attended a study clinic where a range of sociodemographic, clinical, laboratory and HRQOL information was collected by the research nurse. Consenting participants were then contacted by a research pharmacist (WHT) to arrange an additional interview regarding participants’ medications and medication-taking behaviour. At follow-up (at least a year after the baseline assessment), participants attended a clinic for an additional interview (between August 2017 and October 2018). This study was approved by the Tasmanian Health and Medical Human Research Ethics Committee (H0015099).

### 2.2. Measures

#### Medication-Related Factors

Medication-related information collected from participants during the baseline interview was verified using electronic health records. To determine actual medication burden, the validated 65-item medication regimen complexity index (MRCI) [16] and simple medication count were used. Perceived burden of medication (PBM), a tool previously developed and validated in adults with ESKD, was used to assess participants’ perceived burden of their medication regimens [11]. This tool consists of six Likert-scale questions asking if patients feel bothered by the number of medications they take, the size of the pills, adverse effects of medications, the dosing frequency, the need to take medications at work or in social contexts, and the need to drink fluid to take medications.

Medication adherence was self-reported by participants at baseline using the Morisky-Green-Levine Scale (MGLS). [17] This scale consists of four questions with ‘yes/no’ answers, with patients deemed nonadherent if they respond ‘yes’ on at least one of the questions. The Tool for Adherence Behaviour Screening (TABS), a questionnaire developed in Australia to assess adherence behaviour in adults taking chronic medications [18], was also used during the interview. This tool assesses both intentional and unintentional nonadherence, and has two subcomponents: one for ‘adherence’ and one for ‘nonadherence.’ The subcomponents have four items each; a differential score (i.e., total for ‘adherence’ minus total for ‘nonadherence’) of ≥15 reflects good adherence and of ≤14 indicates suboptimal adherence [19].

### 2.3. Covariates

Patient characteristics, including age, gender, marital status, level of education and means of income, and smoking history (current/former vs. never), were recorded at baseline. An index of Socioeconomic Disadvantage was retrieved using the postcode of participants from the Socio-Economic Indexes for Areas of the Australian Bureau of Statistics [20]. The modified Charlson’s Comorbidity Index (CCI) [21], calculated using medical conditions as reported by the participant’s treating physician, was used to determine the medical comorbidities. Baseline laboratory values, including haemoglobin, eGFR and serum creatinine, were extracted using electronic records. eGFR was calculated using the CKD Epidemiology Collaboration (CKD-EPI) equation [22].

Participants self-reported their level of functionality using the basic Activities of Daily Living (ADL) [23] and Instrumental Activities of Daily Living (IADL) [24]. The ADL assesses the ability to perform six basic self-care tasks independently, with scores ranging between 0 and 6 for low to high level of functioning. The IADL assesses functionality to deal with more complex tasks, including the handling of finances and managing medications. This tool contains eight items ranging from 0, for highly dependent individuals, to 8, for those who are increasingly independent. Treating physicians also rated the functionality of the participants using the Karnofsky Performance Scale [25]. Scores range between 0 and 100, with higher scores corresponding to greater performance. Participants self-reported their desire for autonomy using the Autonomy Preference Indexes. This tool includes an eight-item decision-making (preference to be involved in decision-making) and a six-item information-seeking (desire to be informed) scales. Each of these scales were then standardised into scores ranging between 0 and 100 [26]. Zero indicates low preference for autonomy (i.e., delegating to healthcare professionals), while 100 indicates a strong preference and 50 a neutral attitude [26,27]. Cognitive functioning was objectively measured using the Montreal Cognitive Assessment (MOCA), with scores ≥ 26 indicating good cognitive functioning [28]. The Patient Health Questionnaire, a nine-item diagnostic tool, was used to assess the presence of depression [29].

HRQOL was self-reported by participants at baseline and at follow-up using the Kidney Disease Quality of Life Short-Form health survey (KDQOL-36) [30]. This tool consists of a combination of kidney disease-targeted items and generic items. The disease-specific part consists of eleven domains including two dialysis related domains. Therefore, nine domains assessing symptoms, effects, burden, work status, cognitive function, social interaction, sexual function, sleep and social support were applicable to this cohort. Participant responses were transformed into a 100-point scale, with higher scores reflecting better health. The short form (SF-36) is a 36-item questionnaire that assesses eight generic health domains, comprising physical functioning, physical role limitations, pain, general health, vitality, social functioning and mental health. These domains are then aggregated into two component summary scores; physical health (PCS) and mental health component summaries (MCS), with higher scores reflecting greater self-reported HRQOL. Change in HRQOL was examined using the difference between baseline and follow-up MCS and PCS scores, with score differences of ≥5 considered clinically significant [12].

## 3. Statistical Analyses

Variables were checked for normality of distribution via visual inspection of histograms. Normally-distributed continuous variables were reported as mean ± standard deviation (SD), and nonnormally-distributed variables were reported as median (interquartile range [IQR]). Frequency (percentage) was used to report proportions.

Participant characteristics were compared with respect to medication adherence (yes/no). Student’s *t*-test was used to compare continuous variables with a normal distribution, while Mann Whitney-U test was applied for nonnormally distributed variables. Chi-square test was used for comparison of categorical variables. Factors associated with medication nonadherence were examined using binary logistic regression, with effect sizes reported using odds ratios (ORs) and 95% confidence intervals (CIs). Factors were included in the final model based on a *p*-value < 0.1 on univariate analyses or set a priori based on clinical importance and previous research [2,11,12]. The decision-making and information-seeking scales were treated in these analyses both as continuous and categorical variables. A cut-off point of 50 was used for categorising decision-making and information-seeking scales, as this score is considered to show neutrality in terms of increased preference for participation in one’s care as opposed to delegating it to healthcare professionals [26]. To identify factors associated with actual and perceived medication burden, linear regression models were utilised, with associations reported using coefficients (β) and 95% CIs.

Finally, changes in different HQROL measures at baseline and follow-up were compared using a paired *t*-test. Associations between medication nonadherence (MGLS) and changes in different HRQOL measures were performed using linear regression models, with analyses adjusted for age, gender and baseline eGFR. Clinically significant changes in physical (PCS) and mental (MCS) quality of life measures were compared by adherence status using a Chi-square test. The median (IQR) changes in PCS and MCS over the follow-up period are illustrated using boxplots. A *p* < 0.05 was set to determine statistical significance. STATA version 15.1 software (StataCorp LLC, College Station, TX, USA) was used for analysis.

## 4. Results

**Figure 1:**
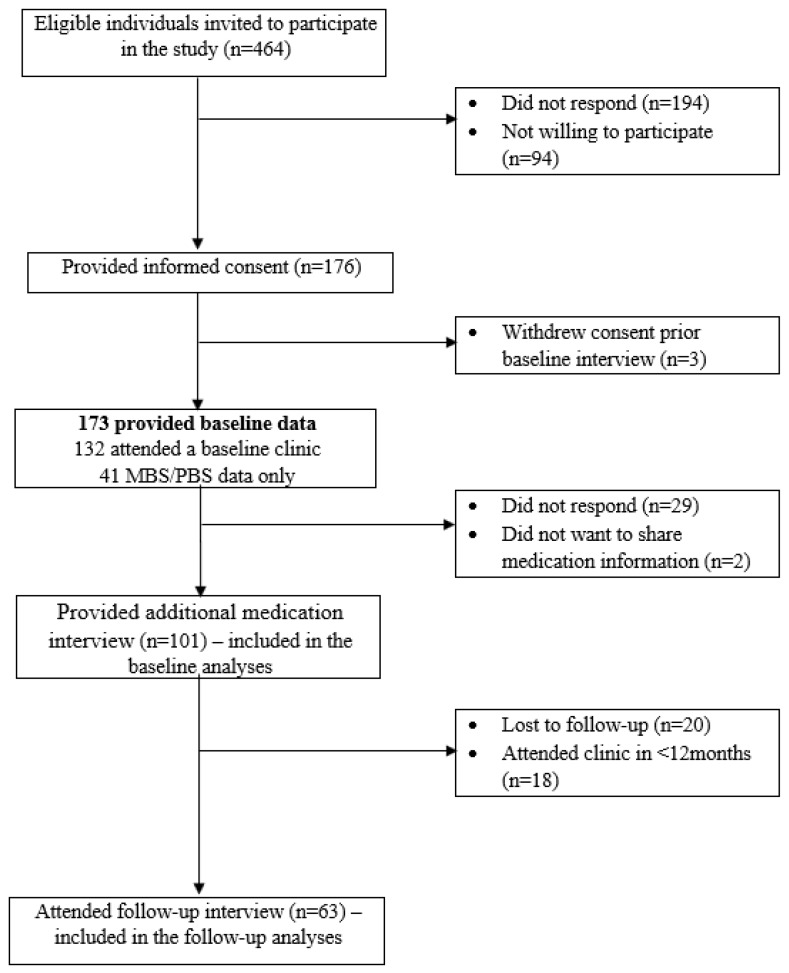
Flow diagram of the recruitment process.

Overall, 464 eligible individuals were invited to participate in the study (Figure 1). Of these, 132 (28%) attended a baseline study clinic appointment and 101 (21%) participated in an additional medication interview. Subsequently, 63 (62%) of these participants completed the follow-up interview. Participants at baseline were predominantly men (67%), with a mean age and eGFR of 72 (SD 11) years and 21 (SD 6) mL/min/1.73 m^2^, respectively. Participants were not different from nonparticipants in terms of age and Index of Socioeconomic Disadvantage (*p* > 0.05). However, a higher proportion of nonparticipants were women (52% vs. 32%, respectively; *p* = 0.04).

Overall, 79% of the participants were taking ≥ 5 medications at baseline and 43% were taking ≥ 9 medications. Based on the MGLS, about 43% of the participants were considered nonadherent, while 60% reported suboptimal adherence based on TABS. API scores revealed that while most participants were interested in having more information (mean API information-seeking; 82 ± 11), they preferred healthcare professionals to make decisions for them (mean API decision-making; 45 ± 17). The baseline characteristics of participants by medication adherence are described in Table 1.

### 4.1. Factors Associated with Medication Nonadherence

Table 2a shows the effect of medication burden and other factors associated with medication nonadherence. People who reported nonadherence were more likely to report higher perceived medication burden (OR 4.89; 95% CI 1.02–23.5; *p* = 0.02) after adjusting for age, gender, eGFR, comorbidity and IADL. Actual medication burden (the number of medications and MRCI) was not associated with nonadherence. People with a high desire for decision-making were 4.6 times more likely to report nonadherence compared with those who prefer to delegate decisions to healthcare professionals (adjusted OR 4.56 95% CI 1.68–12.35). Participants with diabetes were more likely to self-report being adherent (adjusted OR 0.36; 95% CI 0.14–0.89).

We also examined factors associated with suboptimal medication adherence assessed by the TABS (Table 2b). Both actual and perceived medication burden were not related to TABS adherence measurement. However, participants with high BMI (≥30 kg/m^2^) were more likely to be nonadherent compared with those with normal BMI after adjusting for age, gender, CCI and eGFR (OR 3.81; 95% CI 1.01–14.5).

### 4.2. Factors Associated with Perceived and Actual Medication Burden

Given the strong association between perceived medication burden and medication nonadherence, we further explored factors associated with PBM (Table 3a). A higher number of medications (β 0.02; 95% CI 0.01 to 0.04) and MRCI scores (β 0.10; 95% CI 0.03 to 0.15) predicted higher perceived medication burden. Additionally, a more frequent dosing interval was associated with higher perceived burden on adjusted analysis (β 0.02; 95% CI 0.01 to 0.03). After adjustment for gender, eGFR, CCI and IADL, increasing age was associated with lower perceived burden from medications (β −0.01; 95% CI −0.014 to −0.004). An increased desire for decision-making (β 0.02; 95% CI 0.01 to 0.03) and a higher desire for information (β 0.02; 95% CI 0.01 to 0.04) were also associated with higher perceived burden.

To examine whether factors associated with perceived medication burden were different from those affecting actual medication burden, we investigated the correlates of MRCI (Table 3b). As expected, patients with diabetes had a more complex medication regimen (β 7.54; 95% CI 3.43 to 11.6). Lower physical (SF36-PCS) (β −0.43; 95% CI −0.62 to −0.26) and mental HRQOL (SF36-MCS) (β −0.21; 95% CI −0.43 to −0.01) at baseline were associated with higher medication burden (MRCI). An inverse association was also observed between kidney disease-targeted HRQOL scales, such as disease symptoms, burden, work status and effects of the disease and medication burden. 

### 4.3. Changes in HRQOL and Its Association with Medication Nonadherence

The mean ± SD follow-up time for participants who completed the second interview was 433 ± 82 days (~14 months), with no difference in follow-up duration observed between adherent vs nonadherent groups (436 ± 88 vs. 430 ± 77 days; *p* = 0.76)

The changes in different components of HRQOL, both kidney disease-targeted and generic SF-36 scales, are presented in the Appendix A attached. Follow-up data were completed by 63 and 60 participants for kidney-disease targeted scales and generic SF-36 scales, respectively. Out of the disease-targeted components, only the burden of kidney disease showed a significant change at follow-up (mean ± SD score declined from 77 ± 25 to 70 ± 31; *p* = 0.01). Overall, there was no association between medication adherence and changes in kidney disease-targeted scales over time.

Of the 60 participants with completed generic HRQOL data (SF-36), a decline of any magnitude in physical and mental HRQOL was observed in 58% at around 14 months of follow-up. A clinically significant decline in physical HRQOL (change in SF36-PCS ≤ 5) was observed in 35% of participants overall, representing 26% of adherent and 45% of nonadherent participants (*p* = 0.20). A significant reduction in mental HRQOL was also observed in 35% of participants overall, which represented 42% of adherent and 28% of nonadherent individuals (*p* = 0.16).

**Figure 2:**
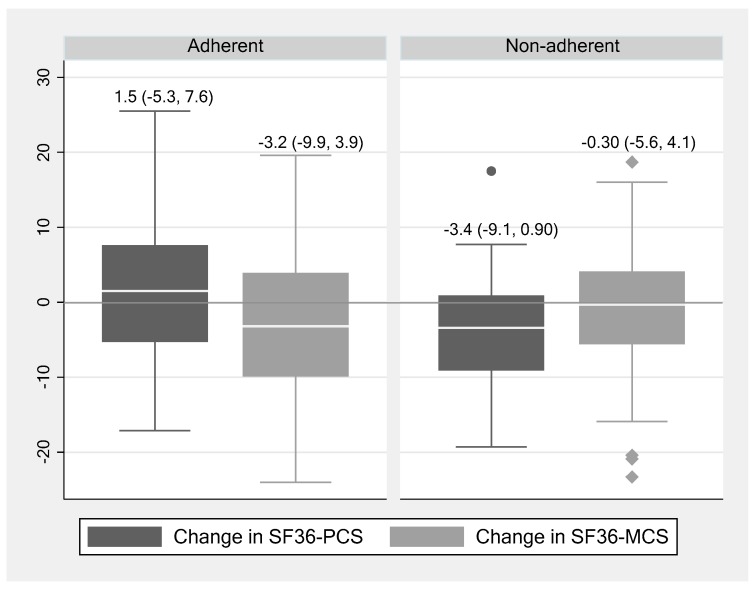
Changes in physical (SF36-PCS) and mental (SF36-MCS) health-related quality of life over 14-months by adherence status (MGLS).

As illustrated in Figure 2, the physical HRQOL was improved over time in adherent individuals compared with a decline in their nonadherent counterparts (a median [IQR] change in PCS of 1.5 [−5.3, 7.6] vs. −3.4 [−9.1, 0.9]; *p* = 0.06). Further, medication nonadherence showed a significant negative association with a change in physical HRQOL after adjusting for age, gender and baseline eGFR (β −4.64; 95% CI −9.10, −0.17) (Table 4). There was no association between medication nonadherence and change in mental HRQOL before or after adjustment for the same variables. 

## 5. Discussion

This study indicates that a considerable proportion of adults with predialysis CKD are nonadherent to their medications. The 43% medication nonadherence (MGLS) was lower than that reported by an Australian study on dialysis patients that used the same questionnaire, where 57% of participants were nonadherent [10]. This is understandable, given the relatively higher degree of medical complexity in dialysis patients compared to those in the earlier stages of CKD [10,31]. Of note, a greater proportion of suboptimal adherence (60%) was identified via the TABS questionnaire. This could relate to the differences in the constructs of the two questionnaires [16,17]. In addition to medication adherence, the TABS, for example, also captures patients’ experiences and behaviour concerning disease management [18]. This shows that medication nonadherence is multidimensional in nature and needs different strategies to detect in patients with predialysis CKD.

Importantly, perceived burden (PBM) of medications, not the actual burden, was associated with medication nonadherence. The association between PBM and nonadherence is in contrast with a prior Australian study that showed no relationship between these factors [10]. A study from Italy showed that perceived burden can modulate the relationship between medication regimen complexity and adherence in dialysis patients [11]. In this study, Neri et al. found that each pill that was added to a regimen of a patient with low PBM was associated with a 5% increase in the odds of nonadherence [11]. This was not the case in those with high PBM, where regimen complexity was not associated with nonadherence [11]. The findings highlight the need to evaluate the perceived burden, alongside actual medication burden, to optimise adherence. Also, simplifying a medication regimen may not effectively improve adherence unless patients’ perceptions are concomitantly addressed [11].

Another interesting result from this study was that people with an increased desire for autonomous decision-making were more likely to be nonadherent. This corresponds with a finding from a study on patients with asthma that applied the same set of questionnaires [32]. The relationship between desire for decision-making and nonadherence could relate to intentional nonadherence, where patients make a conscious decision to skip medications. This phenomenon has been explained by a qualitative review that identified ‘rationalised nonadherence’ as a mechanism used by patients to avoid treatment disruptions of their daily routine [33]. A similar finding was reported in dialysis recipients where people tended to consciously overlook treatments, which the authors termed ‘active nonadherence’ [34]. This is particularly common with medications they considered less important or less easy to adhere to [34]. Therefore, there is a need to foster optimal patient-centred care to actively engage patients in conversations that enable them to acknowledge medication-related difficulties in view of improving adherence [33]. Reiterating the importance of medications in slowing disease progression at the point of care could also help improve adherence. Finally, obese participants (BMI ≥ 30 kg/m^2^) were more likely to be nonadherent than people with normal BMIs based on the TABS. The relationship between higher BMI and poor adherence has been reported in older men previously [35]. This association could be because nonadherence in these individuals might also extend to exercise or dietary restrictions [35].

Highly complex regimens and more frequent dosing were associated with higher perceived medication burden, while older age was associated with feeling lower burden. The association of regimen complexity and more frequent dosing with perceived treatment burden has been reported [36,37]. These factors are important given their practical relevance and the relative ease with which they may be targeted by interventions seeking to reduce medication burden [36]. For instance, the use of long-acting alternatives instead of the repeated use of immediate-release medications is one strategy that can reduce the dosing frequency. Nevertheless, it is important to understand that even less complex regimens could prove burdensome in some patients [37]. Particularly, patients with limited cognitive functionality or with little support could be affected in this regard. The association between older age and lower perceived treatment burden is in line with prior studies [11,36,37]. This may be associated with older people’s adaptation to medications after long-term use [36]. Older adults may also consider their medications more a matter of necessity rather than a burden [36].

Lower HRQOL (kidney disease-targeted and generic scales) were predictive of actual medication burden (MRCI). The association between HRQOL measures and regimen complexity was independent of Charlson’s comorbidity score, suggesting that regimen complexity may capture additional information on the overall disease status of patients [31]. This may also strengthen our prior hypothesis, i.e., that regimen complexity could serve as a proxy measure of overall health in patients with CKD [38]. Also, an inverse relationship between medication burden and HRQOL has been previously reported in predialysis patients with CKD [13].

Medication nonadherence was not associated with baseline HRQOL; however, it was associated with a decline in physical, but not mental, HRQOL (SF36-PCS) over time. This finding was despite the significant decline in mental HRQOL during follow-up for all participants. A study from the AusDiab cohort previously reported that a physical decline in HRQOL is dependent on baseline eGFR values [12]. However, we found no association between baseline eGFR and changes in HRQOL over time.

This study has some strengths and weaknesses. Examining people with advanced CKD not receiving renal replacement therapy adds a new perspective to the literature, as patient-reported medication experiences in this patient group are currently lacking. This is also the first study to examine the association between medication nonadherence and a change in HRQOL over time in patients with CKD. The inclusion of in-depth patient, clinical, medication and social factors is another strength of this study. The relatively small number of participants included may limit the generalisability of the study; however, recruiting people with relatively poorer health and lower functional status is difficult [15]. The use of self-report, but not objective measures, to assess adherence is another limitation of the study, as nonadherent behaviour is often under-reported due to social desirability bias. However, self-reported adherence measures have an advantage in terms of ease of implementation in real practice. In addition, the self-report measures applied in this study capture both intentional and unintentional adherence.

## 6. Conclusions

This study indicates that medication nonadherence is common in adults with predialysis CKD. Perceived medication burden was a predictor of nonadherence, highlighting the need to incorporate patient-reported medication experiences in routine CKD care. Further, while medication regimen complexity was negatively associated with both physical and mental HRQOL at baseline, nonadherence was associated with a decline in physical HRQOL over time. This finding suggests the potential role of medication-related factors in modifying patient-centred outcomes and the need for further research to better understand this relationship.

## Figures and Tables

**Table 1:** Characteristics of participants by medication adherence (*n* = 101).

		Adherence (MGLS)			Adherence (TABS)		
Variables	Total (*n* = 101)	Yes (*n* = 58)	No (*n* = 43)	*p*	Yes (*n* = 40)	No (*n* = 61)	*p*
**Sociodemographic**							
Age (years)	72 (11)	73 (11)	70 (11)	0.12	74 (11)	70 (11)	0.06
Male gender, *n* (%)	68 (67)	36 (53)	32 (47)	0.19	31 (46)	37 (54)	0.08
Level of education (year 12 or less), *n* (%)	63 (62)	38 (60)	25 (40)	0.45	26 (41)	37 (59)	0.66
Married/de facto, *n* (%)	65 (49)	36 (55)	29 (45)	0.58	27 (41)	38 (59)	0.59
Government pension, *n* (%)	64 (63)	37 (58)	27 (42)	0.6	27 (42)	37 (58)	0.67
Index of Disadvantage (highest quartile)	33 (33)	19 (58)	14 (42)	0.3	11 (33)	22 (67)	0.47
Autonomy preference index							
Decision-making	45 (17)	42 (15)	49 (18)	0.04	41 (13)	47 (18)	0.1
Information-seeking	82 (11)	83 (11)	82 (11)	0.78	82 (11)	83 (11)	0.74
Karnofsky performance scale	87 (10)	86 (8)	88 (12)	0.54	87 (9)	86 (12)	0.65
Major depression (PHQ-9 score ≥ 10)	12 (12)	5 (42)	7 (58)	0.24	6 (50)	6 (50)	0.61
**Clinical**							
Smoking (former/current), *n* (%)	51 (50)	29 (57)	22 (43)	0.91	23 (45)	28 (55)	0.25
Comorbidity index, median (IQR)	3 (1–4)	3 (1–5)	2 (1–3)	0.38	3 (2–4)	2 (1–3)	0.04
Common comorbidities, *n* (%)							
Hypertension	90 (94)	52 (58)	38 (42)	0.71	38 (42)	52 (58)	0.67
Diabetes mellitus	39 (40)	28 (72)	11 (28)	0.02	13 (33)	26 (67)	0.19
Atherosclerotic disease	36 (37)	20 (56)	16 (44)	0.74	18 (50)	18 (50)	0.18
Congestive heart failure	17 (18)	7 (41)	10 (59)	0.12	9 (53)	8 (47)	0.27
Peripheral vascular disease	13 (13)	6 (46)	7 (54)	0.37	8 (61.5)	5 (38.5)	0.11
Malignant neoplasm	20 (21)	14 (70)	6 (30)	0.22	13 (65)	7 (35)	0.01
Body mass index, kg/m^2^	30 (6)	31 (6)	30 (5)	0.67	28 (26–31)	32 (27–35)	0.02
ADL	5.8 (0.4)	5.9 (0.3)	5.7 (0.5)	0.1	5.8 (0.4)	5.8 (0.4)	0.36
IADL	5.5 (1.4)	5.5 (1.4)	5.2 (1.1)	0.06	5.4 (1.4)	5.6 (1.5)	0.34
Cognitive impairment (MOCA < 26), *n* (%)	65 (67)	41 (72)	24 (60)	0.22	23 (35)	42 (65)	0.17
**Laboratory**							
Haemoglobin (g/L)	119 (18)	117 (15)	121 (22)	0.3	119 (20)	119 (17)	0.99
Serum creatinine (μmol/L)	265 (112)	249 (101)	288 (122)	0.03	266 (101)	265 (119)	0.97
eGFR (mL/min/1.73 m^2^)	21 (7)	22 (6)	21 (7)	0.51	21 (7)	21 (6.5)	0.89
**Medical**							
No. of medications, median (IQR)	8 (6–11)	8 (6–11)	8 (6–11)	0.73	8 (5–11)	8 (6–10)	0.73
MRCI, median (IQR)	19 (14–27)	20 (9–28)	17 (14–27)	0.41	19 (17–27)	19 (14–27)	0.76
PBM, median (IQR)	1.17 (1–1.33)	1 (1–1.33)	1.33 (1–1.33)	0.01	1 (1–1.33)	1.33 (1–1.33)	0.04
**HRQOL (SF-36)**							
PCS	39 (10)	39 (10)	39 (10)	0.65	39 (10)	39 (10)	0.96
MCS	51 (10)	51 (9)	50 (11)	0.62	51 (10)	51 (10)	0.96

Abbreviations: ADL, activities of daily living; BMI, body mass index; CCI, Charlson’s comorbidity index; eGFR, estimated glomerular filtration rate; IADL, instrumental activities of daily living; IQR, interquartile range; MCS, mental component summary; MGLS, Morisky Green Levine Scale; MOCA, Montreal cognitive assessment; PBM, perceived burden of medication; PCS, physical component summary; PHQ-9, 9-item patient health questionnaire; SD, standard deviation; TABS, Tool for Adherence Behaviour Screening. Results are presented in mean (SD) unless described otherwise.

**Table 2:** Correlates of medication nonadherence.

**a. Nonadherence (MGLS)**	**Unadjusted ORs (95% CIs)**	Adjusted ORs (95% CIs) *
No. of medications	0.97 (0.87–1.08)	0.96 (0.85–1.07)
MRCI (cont.)	0.83 (0.55–1.26)	0.89 (0.56–1.44)
PBM (cont.)	**4.02 (1.03–16)**	**4.89 (1.02–23.5)**
Having diabetes	**0.37 (0.15–0.91)**	**0.36 (0.14–0.89)**
Decision making (cont.)	**1.11 (1.001–1.23)**	**1.15 (1.02–1.29)**
Decision-making (cat; score > 50)	**3.29 (1.41–7.69)**	**4.56 (1.68–12.35)**
**b. Nonadherence (TABS)**	**Unadjusted ORs (95% CIs)**	**Adjusted ORs (95% CIs)**
No. of medications	1.02 (0.91–1.13)	1.04 (0.92–1.18)
MRCI (cont.)	1.003 (0.96–1.05)	1.01 (0.96–1.06)
PBM (cont.)	3.67 (0.84–16.1)	2.78 (0.53–14.5)
BMI (≥30 kg/m^2^)	2.85 (0.21–2.6)	**3.81 (1.01–14.5)**

API, Autonomy preference index; Cat., categorical; CIs, confidence intervals; Cont., continuous; MGLS, Morisky Green Levine Scale; PBM, Perceived burden of medications; ORs, odds ratios. * Analysis adjusted for age, gender, eGFR, Charlson’s comorbidity index and IADL.

**Table 3:** Correlates of perceived and actual medication burden.

**a. Perceived Medication Burden (PBM)—Continuous**		
Variables	Unadjusted β (95% CIs)	Adjusted β (95% CIs)
No. of medications	0.02 (0.01, 0.04)	0.02 (0.01, 0.04)
MRCI (cont.)	0.08 (0.02, 0.14)	0.10 (0.03, 0.15)
Dosage form	0.023 (−0.001, 0.05)	0.024 (−0.001, 0.05)
Dosing frequency	0.02 (0.01, 0.03)	0.02 (0.01, 0.03)
Additional instructions	0.01 (−0.01, 0.02)	0.01 (−0.01, 0.03)
Age (cont.)	−0.01 (−0.014, −0.004)	−0.01 (−0.015, −0.005)
API Decision-making (cont.)	0.02 (0.01, 0.03)	0.02 (0.01, 0.03)
API Information-seeking (cont.)	0.02 (0.01, 0.04)	0.02 (0.01, 0.04)
**b. Actual Medication Burden (MRCI)—Continuous**		
Variables	Unadjusted β (95% CIs)	Adjusted β (95% CIs)
No. of medications	2.44 (2.26, 2.63)	2.49 (2.31, 2.67)
Having diabetes	7.31 (3.57, 11.1)	7.54 (3.43, 11.6)
Kidney disease-targeted scales		
Symptom	−0.26 (−0.38, −0.15)	−0.25 (−0.37, −0.17)
Effects of kidney disease	−0.19 (−0.34, −0.06)	−0.23 (−0.39, −0.08)
Burden of kidney disease	−0.12 (−0.19, −0.04)	−1.64 (−0.25, −0.08)
Work status	−0.09 (−0.15, −0.04)	−0.09 (−0.14, −0.03)
SF-36 generic scales		
MCS	−0.15 (−0.35, 0.04)	−0.21 (−0.43, −0.01)
PCS	−0.43 (−0.61, −0.26)	−0.44 (−0.62, −0.26)

API, autonomy preference index; MRCI, medication regimen complexity index; MCS, mental component summary; PCS, physical component summary; SF-36, 36-item short form survey. Analysis adjusted for age, gender, Charlson’s comorbidity index, activities of daily living (IADL) and cognitive functioning (MOCA).

**Table 4:** Association between medication nonadherence (MGLS) and changes in physical and mental health-related quality of life health over time.

	Unadjusted β (95% CIs)	Adjusted β (95% CIs) *
SF36-PCS		
Nonadherence	−3.99 (−8.29, 0.31)	−4.64 (−9.10, −0.17)
SF36-MCS		
Nonadherence	1.82 (−3.12, 6.78)	2.03 (−2.99, 7.05)

* analyses adjusted for age, gender and baseline eGFR.Abbreviations: CIs, confidence intervals; MGLS, Morisky Green Levine Scale; SF36-PCS, short form physical component summary; SF36-MCS, short form mental component summary; physical component summary.

## Appendix A

This appendix contains the change in different disease-specific and generic components over time, as assessed by KDQOL-36.

**Table A1:** Health-related quality of life (HRQOL) measures at baseline and at 14-months follow-up (KDQOL-SF36), the Tasmanian Chronic Kidney Disease study.

HRQOL Scales	All Participants (*n* = 101)	Participants with Follow-Up Data		
Kidney Disease-Related Scales (*n* = 63)		Baseline	Follow-Up	*p-*Value ***
Burden of kidney disease	75 (24)	77 (25)	70 (31)	0.01
Symptoms	79 (16)	78 (16)	77 (15)	0.59
Cognitive function	81 (16)	81 (15)	79 (16)	0.45
Effect of kidney disease	88 (13)	88 (14)	86 (17)	0.19
Sleep	64 (19)	62 (20)	62 (22)	0.80
Social interaction	82 (15)	82 (15)	82 (15)	0.89
Social support	83 (25)	87 (22)	82 (27)	0.18
Work status	43 (34)	42 (33)	41 (35)	0.64
Overall health status	63 (18)	62 (19)	59 (19)	0.08
**SF-36 scales (*n* = 60)**				
Physical function	56 (26)	56 (26)	55 (30)	0.78
Physical role limitations	47 (42)	49 (39)	36 (38)	0.01
Pain	60 (28)	64 (27)	60 (28)	0.13
General health	49 (29)	46 (20)	44 (21)	0.29
Emotional well-being	79 (22)	77 (18)	76 (19)	0.57
Emotional role limitations	70 (40)	77 (36)	65 (38)	0.02
Social function	77 (30)	77 (26)	68 (29)	0.004
Vitality	55 (27)	48 (23)	45 (24)	0.22
SF36-PCS	38 (10)	38 (11)	37 (11)	0.22
SF36-MCS	51 (11)	52 (11)	49 (11)	0.03

Abbreviations: SF-36, 36-item short form survey; MCS, mental component summary; PCS, physical; component summary; * Paired *t*-test; Results are in mean (SD).
